# Lower bioenergetic costs but similar immune responsiveness under a heat wave in urban compared to rural damselflies

**DOI:** 10.1111/eva.13041

**Published:** 2020-07-09

**Authors:** Nedim Tüzün, Robby Stoks

**Affiliations:** ^1^ Evolutionary Stress Ecology and Ecotoxicology University of Leuven Leuven Belgium

**Keywords:** cellular energy allocation, encapsulation, energy budget, heat stress, heat wave, parasites, stress response, urbanization

## Abstract

There is mounting evidence that the widespread phenotypic changes in response to urbanization may reflect adaptations caused by rapid evolutionary processes driven by urban‐related stressors. Compared to increased habitat fragmentation and pollution, adaptations towards another typical urban‐related stressor, that is higher and longer lasting very high temperatures (heat waves), are much less studied. Notably, the sensitivities to heat waves of life‐history traits and important fitness‐related physiological traits such as immune responsiveness and bioenergetic variables (energy availability, energy consumption and their balance) have never been contrasted between urban and rural populations. By conducting a laboratory common‐garden experiment, we compared effects of a simulated heat wave on life history (survival and growth rate), immune responsiveness and bioenergetic variables between three urban and three rural populations of the damselfly *Coenagrion puella*. Because energy‐mediated trade‐off patterns may only be detected under energetically costly manipulations, all larvae were immune‐challenged by simulating ectoparasitism by water mites. As expected, the simulated heat wave caused negative effects on nearly all response variables. The immune responsiveness, on the other hand, increased under the heat wave, consistent with a trade‐off pattern between immune function and growth, and this similarly between urban and rural populations. A key finding was that urban larvae suffered less from the simulated heat wave compared to the rural larvae in terms of a lower heat wave‐induced depletion in energy availability. This suggests an adaptation of urban populations to better cope with the stronger and more frequent heat waves in cities. Notably, this urbanization‐driven evolution in the bioenergetic variables was not apparent in the absence of a heat wave. Given that changes in energy budgets have strong fitness consequences, our findings suggest that the evolved higher ability to cope with heat waves is fundamental for the survival of urban damselfly populations.

## 1 INTRODUCTION

Animals and plants from a wide range of taxa have undergone adjustments that allow them to cope with novel environmental challenges associated with the ongoing urbanization (Alberti et al., 2017). Habitat fragmentation, pollution and higher temperatures (“urban heat islands”) are amongst the major urban‐related stressors (Parris, 2016). Recent work indicates that the observed phenotypic change in response to urbanization may not just be plastic, but reflect genetic adaptations caused by rapid evolutionary processes driven by these urban‐related stressors (Donihue & Lambert, 2015; Johnson & Munshi‐South, 2017). For example, stronger habitat fragmentation in urban habitats was found to cause evolution towards lower seed dispersal in the weed *Crepis sancta* (Cheptou, Carrue, Rouifed, & Cantarel, 2008), and the higher levels of pollution in urban waters have driven the evolution of a higher tolerance to polychlorinated biphenyls in the killifish *Fundulus heteroclitus* (Whitehead, Pilcher, Champlin, & Nacci, 2012). Distinct biotic selection pressures such as predation and parasitism in urban habitats have also been shown to drive adaptive trait changes, for example in the sexual signalling of a Neotropical frog (Halfwerk et al., 2019).

One particular thermal aspect of cities that likely acts as a strong selective factor is heat waves, that is periods of several days with very high temperatures (Meehl & Tebaldi, 2004). Heat waves have a higher intensity, duration and frequency in urbanized areas (Ward, Lauf, Kleinschmit, & Endlicher, 2016; Zhao et al., 2018), and these heat wave attributes are expected to further increase under global warming (Meehl & Tebaldi, 2004; Wouters et al., 2017). Given that organismal performance of ectotherms rapidly decreases above optimal temperatures (Angilletta, 2009), the very high heat wave temperatures typically have adverse effects on their performance (Feder & Hoffman, 1999; González‐Tokman et al., 2020). Negative effects of heat waves include reduced growth and development rates (e.g., in butterflies: Fischer, Klockmann, & Reim, 2014), lifespan (e.g., in moths: Zhang, Rudolf, & Ma, 2015) and survival (e.g., in damselflies: Van Dinh, Janssens, & Stoks, 2016). Heat waves are therefore potentially powerful selective factors capable of causing adaptive evolutionary shifts (e.g., Rodríguez‐Trelles, Tarrío, & Santos, 2013), and populations can show local adaptation in their responses to heat stress (e.g., Dittmar, Janssen, Kuske, Kurtz, & Scharsack, 2014; Phillips et al., 2016; Van Heerwaarden, Lee, Wegener, Weeks, & Sgró, 2012). Some recent excellent studies convincingly demonstrated that the higher temperatures in cities were associated with the evolution of an increased acute (typically 1–2 hr) tolerance to extreme high temperatures (higher than the ones experienced during heat waves, measured as CTmax) (e.g., in the water flea *Daphnia magna*: Brans et al., 2017; the acorn ant *Temnothorax curvispinosus*: Martin, Chick, Yilmaz, & Diamond, 2019; the anole lizard *Anolis cristatellus*: Campbell‐Staton et al., 2020). Yet, the ability to deal with acute extreme high temperatures as measured by CTmax may not accurately reflect, and even be traded off against, the ability to cope with longer term (i.e., days) exposure to more environmentally realistic very high temperatures, as encountered during heat waves (Castañeda, Rezende, & Santos, 2015; Johansson & Laurila, 2017; Rezende, Castañeda, & Santos, 2014; Truebano, Fenner, Tills, Rundle, & Rezende, 2018). To advance insights in the selective role of heat waves along urbanization gradients, we therefore need studies that explicitly test for differentiation in the responses to heat stress at very high but environmentally realistic temperatures and for longer durations on fitness‐related traits between rural and urban populations. One rare study testing for effects of brief extreme temperatures (i.e., “thermal spikes”) during the embryonic stage of urban and rural populations of an anole lizard found no evidence for adaptation to the more extreme urban thermal conditions (Hall & Warner, 2018).

Besides life history, several physiological variables can be important fitness‐related traits that may not only underlie stressor effects on life history and performance, but also be more sensitive to stressors (González‐Tokman et al., 2020; Hofmann & Todgham, 2010; Karl, Stoks, De Block, Janowitz, & Fischer, 2011; Sokolova, 2013). Several key fitness‐related physiological variables have been shown to be impaired by heat waves, and in other studies been associated with urbanization, yet have never been integrated in a study directly comparing their sensitivity to heat waves between urban and rural populations. One such candidate fitness‐related physiological variable that has been often studied in an urbanization context is immune responsiveness. Heat waves have indeed been shown to impair immune responsiveness in a variety of taxa (e.g., pond snails: Seppälä & Jokela, 2011; butterflies: Fischer et al., 2014), and urban areas have often been associated with increased parasite abundance and immune responsiveness (reviewed in Isaksson, 2015; Murray et al., 2019, but see, for example Sepp, Mcgraw, Kaasik, & Giraudeau, 2018). Yet, it remains to be tested whether the negative effects of heat waves on immune responsiveness differ between rural and urban populations.

Another set of key fitness‐related physiological variables are bioenergetic variables (Goodchild, Simpson, Minghetti, & DuRant, 2019; Sokolova, 2013). Energy availability and energy consumption are especially important, as these provide information about the “energy gain and loss” balance (Sokolova, 2013). When stressors reduce energy budgets and increase energy consumption, the resulting energy imbalances will affect fitness components, including decreased growth, survival and reproduction (Sokolova, 2013; Verheyen & Stoks, 2020). Exposure to heat stress has been shown to negatively impact bioenergetic variables (e.g., Aurélio et al., 2013; Gandar et al., 2017; Klepsatel, Wildridge, & Gáliková, 2019; Kühnhold et al., 2017). Moreover, both energy storage (e.g., Brans, Stoks, & De Meester, 2018) and energy consumption (e.g., Welbers et al., 2017) have been shown to differ between rural and urban populations. Nevertheless, we lack information on whether urban populations have a higher ability to cope with heat waves in terms of lower bioenergetic costs.

We here compared effects of a simulated heat wave on a set of fitness‐related traits including life history (survival and growth rate), immune responsiveness and bioenergetic variables between three urban and three rural populations of the damselfly *Coenagrion puella*. We have shown before that compared to rural populations, urban populations of *C. puella* evolved differences both in the early larval stage (lower growth rates, Tüzün, Op de Beeck, Brans, Janssens, & Stoks, 2017) and in the adult stage (higher flight performance, Tüzün, Op de Beeck, & Stoks, 2017), and we now extend this to physiological responses to heat waves. We carried out a laboratory common‐garden rearing experiment from the egg stage where half of the larvae per urbanization type experienced a simulated heat wave in their final instar. All larvae were immune‐challenged by simulating ectoparasitism by water mites in order to test for differences in immune responsiveness, and how this is modulated by a heat wave between urban and rural populations. This is a realistic scenario, as water mite parasitism can be high (up to 100%) in natural populations of *C. puella* (Rolff, 2000). Moreover, energetically costly manipulations such as an immune challenge may increase the probability of detecting energy‐mediated trade‐off patterns (Karl et al., 2011; Reznick, Nunney, & Tessier, 2000), hence differentiation between rural and urban populations. Common‐garden experiments coupled with relevant environmental manipulations (i.e., a heat wave and ectoparasites) have been suggested as an ideal approach to identify genetically based phenotypic divergence across urbanization gradients (Rivkin et al., 2019).

We predicted negative effects of the heat wave on the already‐stressed (i.e., immune‐challenged) larvae. For example, larvae of *C. puella* exposed to another energetically costly stressor (a pesticide) showed lower survival when additionally exposed to a heat wave, compared to pesticide‐exposed larvae at control temperatures (Van Dinh et al., 2016). Given that urban areas show more frequent and intense heat waves than rural areas (Ward et al., 2016; Zhao et al., 2018; for the study region: Lauwaet, De Ridder, Maiheu, Hooyberghs, & Lefebre, 2018), we predicted urban damselflies to have evolved a higher ability to cope with heat wave temperatures in the form of a lower bioenergetic cost and a lower reduction in immune responsiveness to the heat wave treatment.

## 2 MATERIALS AND METHODS

### 2.1 Study populations and rearing conditions

**FIGURE 1:**
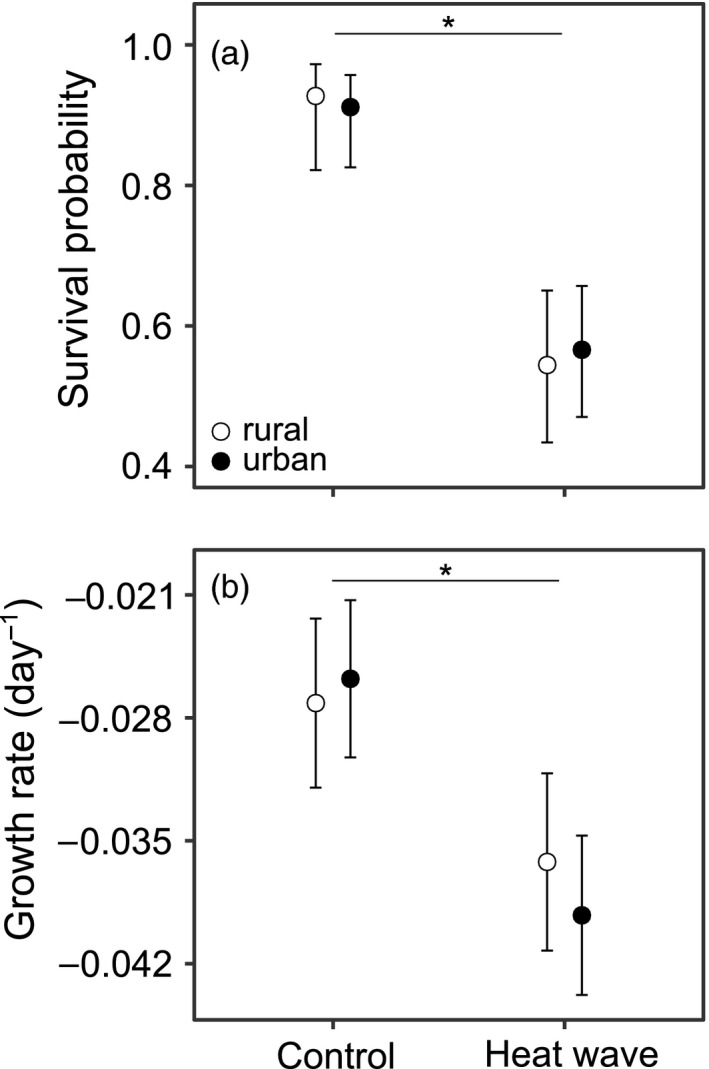
Life‐history responses of immune‐challenged *Coenagrion puella* larvae during the heat wave period, as a function of the heat wave treatment and urbanization level. Least‐square means with 95% confidence intervals are given for (a) survival probability and (b) growth rate. Asterisks indicate significant differences between the control and heat wave treatment (*p* < .05)

*Coenagrion puella* is a common species across large parts of Europe in both urban and rural habitats (Goertzen & Suhling, 2013). The species is univoltine with the adult flight period in late spring and summer during which eggs are being laid. Eggs hatch after a couple of weeks, and larvae emerge the following spring. We studied three populations situated in rural areas (Bierbeek, Bornem, Houwaart), and three populations in urban areas (Leuven, Mechelen, Oudenaarde), all located within a 45 km radius in Flanders, Belgium (see Figure 1 in Tüzün, Op de Beeck, & Stoks, 2017). Cities in Flanders are characterized by a systematically higher number of “heat wave degree days” (a composite parameter accounting for both the duration and the intensity of a heat episode), with an average of 13 heat wave degree days in rural areas compared to 21 heat wave degree days in urban areas recorded for the period 2000–2016 (Lauwaet et al., 2018). Moreover, climate model projections predict this urban–rural difference in the number of heat wave degree days to be further exacerbated (~2×) with future climate change (Wouters et al., 2017). Urban ponds in the study region have a ca. 3°C higher mean summer temperature, and a ca. 4°C higher maximum summer temperature compared to rural ponds (Brans, Engelen, Souffreau, & De Meester, 2018). Moreover, daily temperatures fluctuate ca. 2°C more in urban than in rural ponds (Brans, Engelen, et al., 2018).

A population was classified as rural or urban if the built‐up area surrounding the pond (determined using GIS data) was less than 3% or more than 15%, respectively. The built‐up area criterion excluded roads, pavements and parking lots; hence, a built‐up area higher than 10% refers to highly urbanized areas (Piano et al., 2017). This classification was carried out in two steps. We initially determined three rural and three urban plots of 3 × 3 km, followed by a selection of subplots (200 × 200 m) with the same urbanization level as the plots. This procedure ensures similar urbanization levels at both the local (subplot) and regional (plot) scales (see Figure 1 in Piano et al., 2017 for additional details). Using this sampling design, differentiation in evolutionary and plastic responses between urban and rural populations has been demonstrated for the study species (e.g., Tüzün, Op de Beeck, Brans, et al., 2017; Tüzün, Op de Beeck, & Stoks, 2017; Tüzün & Stoks, 2018), and for other taxa (e.g., Brans, Stoks, et al., 2018; Merckx, Kaiser, & Van Dyck, 2018).

We collected ~15 mated females from each of the six ponds in the period 19–22 June 2016. Females were provided with wet filter papers for oviposition. After hatching, larvae were housed individually in 200‐mL plastic cups filled with dechlorinated tap water and kept at 21°C with a photoperiod of 14:10 hr light:dark. Larvae were fed *Artemia* nauplii five days a week. For the experiment, we focused on the final instar as during this instar the largest mass increase occurs. Moreover, effects experienced in the final larval instar most strongly affect the adult stage (Stoks & Córdoba‐Aguilar, 2012).

We additionally recorded the prevalence of parasitism by water mites of rural and urban adult damselflies. Details of the methods and the associated results are reported in Appendix S1.

### 2.2 Experimental design

To test the effects of a heat wave on immune‐challenged larvae, and whether this differed between urban and rural damselfly populations, we setup an experiment where we crossed urbanization level (urban versus rural) and a heat wave treatment (present versus absent). Note that to address our research questions, it was not needed to consider the immune challenge as a separate experimental factor, yet it was needed to quantify the immune responsiveness as a response variable in all larvae. Adding nonimmune‐challenged larvae to our experimental design would not have added insight in the strength of the immune responsiveness, as the here studied encapsulation response can only be measured on the implanted nylon filaments, hence not in larvae that were not implanted. We therefore immune‐challenged all larvae (see e.g., Duong & McCauley, 2016). Given the higher mortality in the heat wave‐exposed larvae compared to the control group (see Results), we tried to keep the sample size of the heat wave and control groups similar by starting more larvae in the heat wave treatment. This resulted in following initial (final) sample sizes: control‐rural = 55 (50), control‐urban = 79 (70), heat wave‐rural = 79 (43), and heat wave‐urban = 106 (59) (total initial sample size: 319 larvae, total final sample size: 222 larvae). Larvae of each population of the same urbanization level were distributed as equally as possible between the heat wave treatments.

After moulting into their final instar, larvae were randomly allocated to either the heat wave or the control treatments. Larvae assigned to the heat wave treatment were exposed to gradually increasing temperatures during a 4‐day period. The first day, individual cups with larvae were moved to a water bath at 26°C (mean ± *SD*: 25.8 ± 0.3°C). The second day, after the insertion of nylon filaments (see below), cups were transferred to a water bath at 32°C (32.6 ± 0.4°C), where they were kept for the next three days. This thermal regime mimics a typical heat wave (Meehl & Tebaldi, 2004) and corresponds to the heat wave definition by the Belgian Federal Public Service Health (a period of three consecutive days with an average min. temperature ≥ 18.2°C and an average max. temperature ≥ 29.6°C; Lauwaet et al., 2018). In shallow water bodies that are typically occupied by the study species, water temperatures are highly correlated with air temperatures (e.g., Ali, Mishra, Islam, & Alam, 2016; Prapaiwong & Boyd, 2012). Note that water temperatures up to 34°C have been reported in ponds in the study region (Brans, Engelen, et al., 2018). Larvae assigned to the control group were kept at 21°C (21.5 ± 1.1°C) for the same 4‐day period. All larvae were fed daily during the heat wave period (including the control group).

To challenge the immune system, we inserted a nylon filament into the body of the larvae, which is encapsulated via melanin deposition by the damselfly as part of its immune response. This method mimics an immune challenge caused by ectoparasites (e.g., water mites) and has been successfully applied on adult damselflies (e.g., Debecker, Sommaruga, Maes, & Stoks, 2015; Ilvonen & Suhonen, 2016; Rantala, Honkavaara, & Suhonen, 2010), as well as on dragonfly larvae (Duong & McCauley, 2016; Moore, Lis, & Martin, 2018). On the second day of the heat wave (or control) treatment, we inserted sterile filaments (mean length ± *SE*: 1.37 ± 0.03 mm; diameter: 0.25 mm) into the second abdominal pleura on the dorsal side of the sternal–tergal margin of each larva. The filament, knotted at one end, was rubbed with sandpaper to increase the encapsulation area. To facilitate a smooth insertion, we punctured a hole in the cuticle using a syringe needle (0.3 mm diameter) prior to the filament insertion. At the end of the 4‐day heat wave period, that is the end of the experiment, filaments were carefully retrieved. Larvae were stored at –80°C for further physiological analyses, while filaments were stored at –20°C until melanin quantification (see below).

### 2.3 Response variables

We checked daily for survival during the heat wave period. We calculated growth rate during the 3‐day encapsulation period as [ln(final mass) − ln (initial mass)]/3. Initial mass was measured directly before filament insertion, whereas final mass was measured at the end of the experimental period, after removing the nylon filament from the abdomen. We weighed larval wet mass to the nearest 0.01 mg using an electronic balance (AB135‐S, Mettler Toledo) after gently blotting the larvae dry on tissue paper.

We quantified the encapsulation rate by calculating the amount of melanin on the filaments following Rantala and Roff (2007). For this, using a camera attached to a microscope (Olympus BX51) at a magnification of 40×, we photographed the filaments from four different angles (encapsulation on the filaments was not uniform). Next, we measured the encapsulation intensity by quantifying the grey values on the filament with the software Image‐Pro Plus. For each filament, we subtracted the mean grey value (across the four photographs) from the grey value of three blank filaments that were not inserted into a larva. Higher (darker) grey values correspond to higher encapsulation rates.

We assayed the following bioenergetic response variables: levels of storage molecules (fat, protein and sugar contents) and, as measure of metabolic rate, the activity of the electron transport system. All variables were measured on the body supernatants using spectrophotometry based on established protocols for damselfly larvae (Van Dievel, Janssens, & Stoks, 2019; Verheyen & Stoks, 2020). Initially, larvae were homogenized in phosphate‐buffered saline (50 mmol/L, pH 7.4, 90% of the final mass × 15 ml) and centrifuged to obtain the supernatant. Next, total fat content was measured based on a protocol of Marsh and Weinstein (1966), total protein content was quantified by using the Bradford (1976) method, and total sugar content was measured using a protocol based on the glucose kit of Sigma‐Aldrich USA (Stoks, De Block, & McPeek, 2006). We measured the activity of the electron transport system (ETS) to assess oxygen consumption following the protocol of De Coen and Janssen (2003), modified for damselflies (Janssens & Stoks, 2013). We provide the detailed protocols of the bioenergetic response variables in Appendix S2.

Based on the assayed bioenergetic response variables, we quantified the cellular energy allocation (CEA) of each larva as a measure of the net energy budget (De Coen & Janssen, 2003). We calculated CEA as the ratio of energy availability to energy consumption: CEA = energy availability/ energy consumption (Pestana, Loureiro, Baird, & Soares, 2009; Van Dievel et al., 2019; Verheyen & Stoks, 2020; Verslycke, Roast, Widdows, Jones, & Janssen, 2004). Energy availability per larva was calculated as the sum of the energy present in fat, proteins and sugars, by converting these into energetic equivalents using their respective energy of combustion: 39.5 J/mg fat, 24 J/mg protein and 17.5 J/mg glycogen (De Coen & Janssen, 2003). Energy consumption per larva was calculated by converting the total consumed oxygen into energetic equivalents by using the oxyenthalpic equivalent for an average fat, protein and sugar mixture of 0.484 J/mol O_2_ (De Coen & Janssen, 2003).

### 2.4 Statistical analyses

We tested for effects of the heat wave treatment, urbanization level and their interaction on life history, the encapsulation response and bioenergetic variables with separate (generalized) linear mixed‐effect models (GLMM). The survival model was fit using the logit link and a binomial error structure, whereas all other models were fit using the identity link function with a normal error structure. All models included the heat wave treatment, urbanization level and their interaction as fixed effects. Population (nested within urbanization level) was included as a random effect, thereby accounting for variation across populations within an urbanization level. Given that we used multiple larvae per mother, we included the mother identity as an additional random effect. We additionally included body mass (after the encapsulation period) and the age of the larvae as (log‐transformed) covariates to all models (expect for the growth rate model, where only age was included), in order to account for mass‐ and age‐related variation in traits. Significant interactions were further investigated using linear contrasts. To test for trade‐off patterns at the individual level, we used Pearson's correlations to report pairwise relationships between growth rate, the encapsulation response and bioenergetic variables (result reported in Table S1, Appendix S3).

To improve model fits, energy availability and cellular energy allocation were Box–Cox transformed. All analyses were conducted in R v.3.5.3. (R Core Team, 2019), using the packages “*lme4”* for running mixed‐effect models (Bates, Mächler, Bolker, & Walker, 2015), “*car”* for ANOVA tables with *F*‐tests and *p*‐values using the Kenward–Roger approximation (Fox & Weisberg, 2011), and “*emmeans*” for running post hoc tests. Given the slightly unbalanced dataset, models were fit using the restricted maximum likelihood approach (Bates et al., 2015). Model fits were confirmed by visually inspecting the distribution of residuals.

Note that we only report effects of the heat wave, urbanization level and their interaction in the Results section; complete results of all statistical models (including effects of covariates) are provided in Table S2 (Appendix S3). Results for the individual categories of energy storage molecules, that is fat, protein and sugar contents, are reported in Table S3 (Appendix S3), as we keep the focus of the manuscript on (composite) energetic equivalents of these storage molecules, that is energy availability, energy consumption and cellular energy allocation (CEA).

## 3 RESULTS

### 3.1 Larval life history

Larval survival in the heat wave treatment (~56%) was lower than in the control treatment (~92%) (GLMM, χ^2^_1_ = 39.16, *p* < .001, Figure 1a). Survival did not depend on the urbanization level (χ^2^_1_ = 0.02, *p* = .896), or on the interaction between the heat wave treatment and urbanization level (χ^2^_1_ = 0.18, *p* = .673). All (immune‐challenged) larvae showed negative growth rates during the experiment (Figure 1b). These growth rates were ~42% lower in the heat wave treatment compared to the control (LMM, *F*_1,207.4_ = 28.44, *p* < .001). The urbanization level of the larvae (*F*_1,4.1_ = 0.09, *p* = .776), or the interaction effect between the heat wave treatment and urbanization level (*F*_1,208.4_ = 1.02, *p* = .317), did not affect growth rates.

### 3.2 Immune responsiveness

**FIGURE 2:**
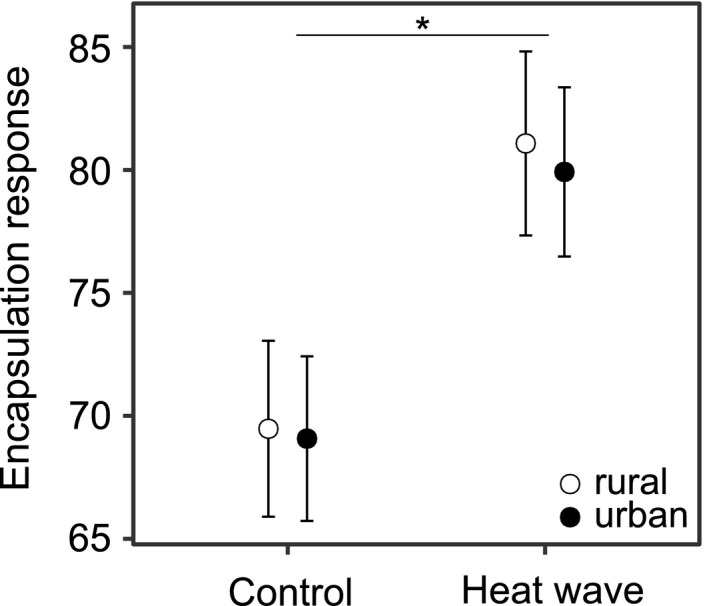
The encapsulation rate of immune‐challenged *Coenagrion puella* larvae as a function of the heat wave treatment and urbanization level. The encapsulation rate is expressed as greyscale values on the inserted filament, with higher (darker) grey values corresponding to higher encapsulation rates. Given are least‐square means with 95% confidence intervals. Asterisks indicates a significant difference between the control and heat wave treatment (*p* < .05)

The encapsulation response, representing the immune reaction to the filament insertion, was ~16% stronger in the heat wave treatment compared to the control treatment (*F*_1,200.1_ = 47.45, *p* < .001; Figure 2). The urbanization level of the larvae (*F*_1,4.1_ = 0.56, *p* = .494), or the interaction effect between the heat wave treatment and urbanization level (*F*_1,202.6_ = 0.06, *p* = .802), did not affect the encapsulation response.

### 3.3 Bioenergetic responses

**FIGURE 3:**
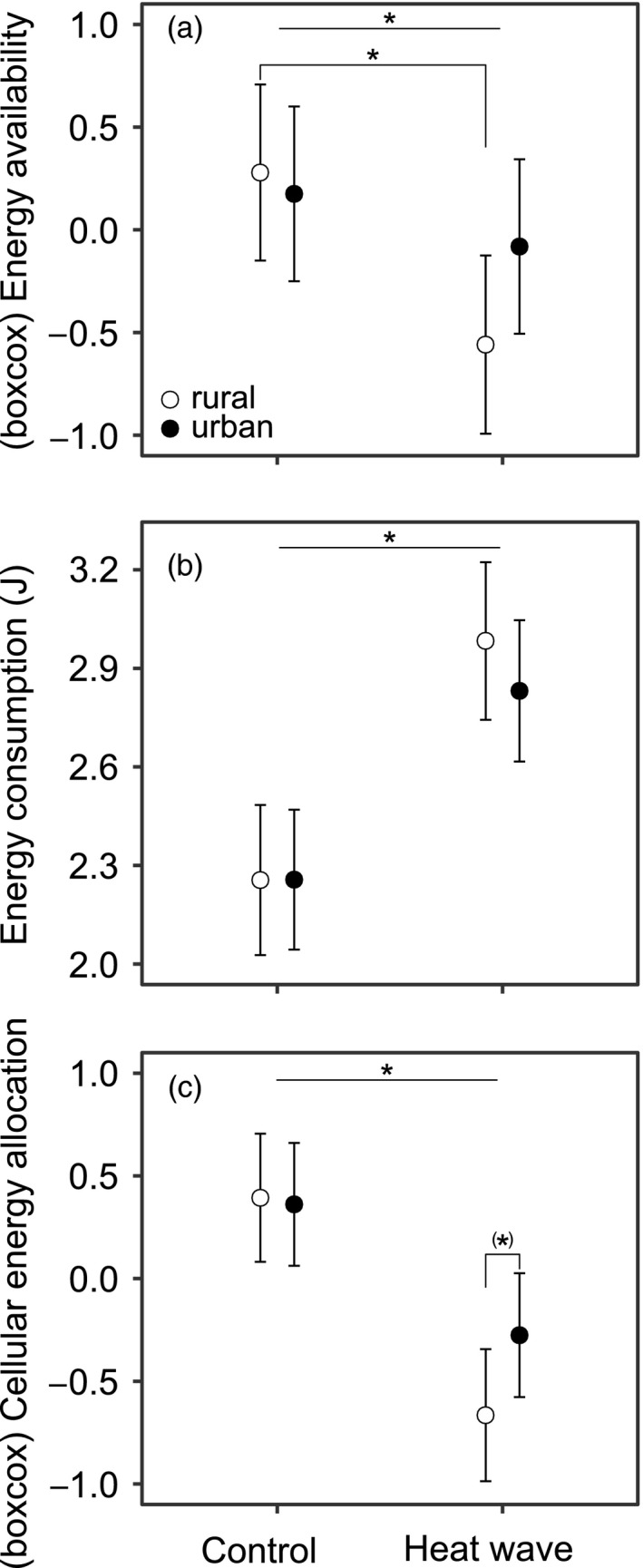
Bioenergetic responses of immune‐challenged *Coenagrion puella* larvae as a function of the heat wave treatment and urbanization level. Least‐square means with 95% confidence intervals are given for (a) energy availability (Box–Cox transformed), (b) energy consumption and (c) cellular energy allocation (Box–Cox transformed). The energy availability was calculated as the sum of the energy present in fat, proteins and sugars, by converting these into energetic equivalents. Energy consumption was calculated by converting the total consumed oxygen (based on ETS activity) into energetic equivalents. Cellular energy allocation was calculated as the ratio of energy availability to energy consumption (see text for further details). Asterisks indicate significant differences between the control and heat wave treatment, as well as between other treatment combinations based on linear contrasts (*p* < .05) (*p* = .079 for the asterisk between parentheses)

The heat wave treatment caused on average a decrease in energy availability (*F*_1,204.9_ = 15.01, *p* < .001), yet this was dependent on the urbanization level of the larvae (Heat wave × Urbanization level, *F*_1,206.1_ = 4.99, *p* = .026; Figure 3a). While rural larvae in the heat wave treatment had ~21% lower energy availability compared to the control group (contrast test, control versus heat wave: estimate ± *SE* = 0.838 ± 0.199, *p* < .001), energy availability in urban larvae did not significantly change with the heat wave treatment (estimate ± *SE* = 0.256 ± 0.168, *p* = .130). The energy consumption was ~30% higher in the heat wave treatment compared to the controls (*F*_1,206.1_ = 37.65, *p* < .001). The urbanization level of the larvae (*F*_1,4.1_ = 0.44, *p* = .545), or the interaction effect between the heat wave treatment and urbanization level (*F*_1,207.6_ = 0.53, *p* = .467), did not affect the energy consumption (Figure 3b). Both rural and urban larvae showed a decrease in cellular energy allocation (CEA) when exposed to the heat wave (*F*_1,205.8_ = 44.62, *p* < .001), yet there was a (nonsignificant) trend for this drop to be stronger in rural (~42%) than in urban (~24%) larvae (heat wave × urbanization level, *F*_1,206.8_ = 2.94, *p* = .088; Figure 3c): while the rural and urban larvae showed similar CEA under control conditions (rural versus urban: estimate ± *SE* = 0.032 ± 0.193, *p* = .872), rural larvae had ~38% lower CEA than urban larvae when exposed to the heat wave (estimate ± *SE* = −0.389 ± 0.203, *p* = .079). [Correction added on 22 July 2020, after first online publication: The figure’s position were changed.]

## 4 DISCUSSION

We showed strong effects of a simulated heat wave on life history, immune responsiveness and bioenergetic variables of immune‐challenged damselfly larvae. While the heat wave effect on the majority of traits was, as expected, negative (i.e., decreased survival, growth rate, and energy availability, and increased energy consumption), the immune response measured as encapsulation rate increased under the heat wave. Notably, we found support for an urbanization‐dependent reaction to the heat wave in terms of energy budget. As expected, urban larvae apparently suffered less from the simulated heat wave compared to the rural larvae, indicated by the stronger heat wave‐induced decrease in energy availability in rural larvae, which also resulted in (a nonsignificant trend for) lower cellular energy allocation (CEA) in rural than urban larvae under the heat wave. As we started the common‐garden experiment from the egg stage, we cannot fully exclude maternal effects contributing to the urban–rural differentiation. In support of these differences being at least partly genetic, maternal effects have been shown to play only a minor role in shaping life‐history traits in coenagrionid damselflies (Shama, Campero‐Paz, Wegner, De Block, & Stoks, 2011; Strobbe & Stoks, 2004). Moreover, egg and hatchling sizes, a common mode of the transfer of maternal effects (Mousseau & Dingle, 1991), do not differ between rural and urban populations of the study species (Appendix S2 in Tüzün, Op de Beeck, Brans, et al., 2017).

### 4.1 General responses to the heat wave

The very high temperatures as experienced during heat waves can have strong negative effects on the fitness of organisms (Colinet, Sinclair, Vernon, & Renault, 2015; Stillman, 2019; Vázquez, Gianoli, Morris, & Bozinovic, 2017). This effect is, amongst others, attributed to thermodynamic rules that control enzyme stability and cell membrane properties, as well as impaired oxygen supply at high temperatures (Hofmann & Todgham, 2010; Kingsolver, 2009; Stillman, 2019). Sensitivity to extreme temperatures can be further exacerbated when the organism is exposed to an additional stressor (e.g., food shortage: Van Dinh et al., 2016), as coping strategies become energy limited. The here reported negative effects of the heat wave on the life‐history traits, that is decreased growth and survival, of immune‐challenged damselfly larvae suggest that the combination of heat wave temperatures and the immune challenge was beyond the larvae's coping capacity.

Exposure to very high temperatures often causes a decrease in immune responses, typically attributed to a reduced energy storage (e.g., Fischer et al., 2014; Janssens, Dinh Van, & Stoks, 2014; Prokkola, Roff, Kärkkäinen, Krams, & Rantala, 2013; Seppälä & Jokela, 2011). Yet, this response is not general, and also increased immune responsiveness under heat stress has been observed (Dittmar et al., 2014; Silva & Elliot, 2016; Wojda & Jakubowicz, 2007). In line with the latter studies, and despite the reduced available net energy, the encapsulation response was stronger under the heat wave. This resembles results from another damselfly, *Ischnura elegans*, where exposure to a heat wave caused an increase in phenoloxidase activity (albeit no immune challenge was given), an enzyme involved in the synthesis of melanin used in the encapsulation response (Arambourou & Stoks, 2015; Van Dievel, Stoks, & Janssens, 2017). The cricket *Gryllus texensis* also showed increased activity in immune‐related (phenoloxidase and lysozyme‐like) enzymes under a simulated heat wave, possibly driven by the increased energy availability due to the higher feeding rates reported at the heat wave temperatures (Adamo & Lovett, 2011). Yet, the reduced growth rate and energy availability under the heat wave in the current study suggests a different mechanism for increased immune responsiveness under stressful temperatures. We propose that the already diminished available energy was allocated to the encapsulation response, away from other processes such as growth. In line with this, we observed a negative correlation between the encapsulation response and growth rate (Table S1, Appendix S3), reflecting a well‐known trade‐off pattern between immune function and growth in damselflies (De Block & Stoks, 2008; Stoks et al., 2006), as well as in other taxa (e.g., Busso, Blanckenhorn, & González‐Tokman, 2017). Other stress‐induced defence mechanisms, such as the upregulation of heat shock proteins, could have further contributed to the increased immune responsiveness (Wojda, 2017). While indeed several studies suggest increased immune responsiveness under heat stress via the expression of heat shock proteins (e.g., the moth *Galleria mellonella*, Wojda & Jakubowicz, 2007; the moth *Mamestra brassicae*, Richards, Dani, Lu, Butt, & Weaver, 2017), an upregulation of heat shock proteins can also be associated with a reduced immune function (e.g., the Pied Flycatcher *Ficedula hypoleuca*, Morales et al., 2006; the butterfly *Lycaena tityrus*, Fischer, Kölzow, Höltje, & Karl, 2011). The few studies reporting a negative correlation between the energetically costly upregulation heat shock proteins and growth rates (overview in Sørensen, Kristensen, & Loeschcke, 2003; in the damselfly *Ischnura elegans*: Stoks & De Block, 2011) suggest that such upregulation might have contributed to the here documented stress‐induced reduced energy availability and growth rate. Yet, validating this conjecture will require explicit measurement of heat shock protein levels.

Despite ad libitum feeding during the heat wave period, energy availability of damselfly larvae decreased under the heat wave, suggesting reduced feeding and/or reduced conversion of ingested food into energy storage (e.g., Adamo & Lovett, 2011; Van Dievel et al., 2017). This reduced energy availability was mainly driven by a decrease in fat content (see Table S3, Appendix S3), a response shown in other insects (e.g., butterflies: Fischer et al., 2014; fruit flies: Klepsatel et al., 2019). The accompanying strong increase in energy consumption (i.e., metabolic rate) under the heat wave (see also e.g., Aurélio et al., 2013; Van Dievel et al., 2017) further points to the increased energy demand. Reduced growth and increased metabolic rate under the heat wave may be explained by food intake not being able to compensate the increase in metabolic demand (e.g., Lemoine & Burkepile, 2012; Rall, Vucic‐Pestic, Ehnes, Emmerson, & Brose, 2010). This mismatch may have resulted in the here reported overall decrease in the available net energy (i.e., CEA) under the heat wave; a pattern matching previous studies in other taxa (e.g., goldfish: Gandar et al., 2017; sea cucumbers: Kühnhold et al., 2017).

### 4.2 Urbanization‐dependent responses to the heat wave

The more stressful conditions associated with urban areas, including chronic human disturbances, higher levels of pollution and higher extreme temperatures (Isaksson, 2015; Murray et al., 2019; Parris, 2016; Ward et al., 2016), are expected to drive adaptations of physiological stress responses in urban populations (e.g., Atwell et al., 2012; Brans, Stoks, et al., 2018; Partecke, Schwabl, & Gwinner, 2006). Yet, work on nonheat wave related stress responses (typically measured via glucocorticoid hormones, the primary stress hormone of vertebrates and strongly linked to the energetic status of the organism; Dantzer, Fletcher, Boonstra, & Sheriff, 2014; Landys, Ramenofsky, & Wingfield, 2006) suggests that urbanization‐related patterns are not consistent (Birnie‐Gauvin, Peiman, Gallagher, de Bruijn, & Cooke, 2016; Bonier, 2012; French, Webb, Hudson, & Virgin, 2018; Sepp et al., 2018). Recent work on the European blackbird, for example, found lower feather corticosterone levels in field‐collected birds from urban populations, but no difference was reported for other stress‐related parameters such as the heterophils/lymphocytes ratio or heat shock proteins, suggesting that overall urban habitats are not physiologically more stressful for this species (Ibáñez‐Álamo et al., 2020). In the only other study comparing heat shock protein levels in an urbanization context, mussels from an urban river site had higher levels of the heat shock protein 70 (HSP70) when compared to mussels from a rural river site, possibly as a response to the higher chemical pollution in the urban site (Ravaschiere et al., 2017).

Parasite abundance and immune responsiveness in vertebrates have been shown to be often higher in more urbanized areas (reviewed in Isaksson, 2015; Murray et al., 2019), yet many exceptions exist (e.g., Bradley & Altizer, 2007; Sepp et al., 2018). In our study, this pattern was only poorly supported by a slightly higher prevalence of water mites, a key ectoparasite of damselflies (Rolff, 2000), in urban adult *C. puella* damselflies (Appendix S1). This also was not associated with a higher encapsulation response in urban populations, neither in the absence nor in the presence of a heat wave. Work on bees revealed a similar pattern: parasite pressure increased with urbanization (Theodorou et al., 2016; Youngsteadt, Appler, López‐Uribe, Tarpy, & Frank, 2015; see also Majewska, Satterfield, Harrison, Altizer, & Hepinstall‐Cymerman, 2019 for nonmigratory monarch butterflies), yet no effects of urbanization on immune responses were detected (encapsulation and phenoloxidase activity: Appler, Frank, & Tarpy, 2015, immune gene expression: Youngsteadt et al., 2015).

In line with the urban heat islands (Chown & Duffy, 2015), a higher acute tolerance to extreme temperatures (measured as CTmax) has been documented in urban populations of ectotherms (e.g., the water flea *Daphnia magna*: Brans et al., 2017; the acorn ant *Temnothorax curvispinosus*: Martin et al., 2019; the anole lizard *Anolis cristatellus*: Campbell‐Staton et al., 2020). Yet surprisingly, almost no study directly tested for a higher ability of urban populations to cope with more realistic chronic exposure to very high temperatures as encountered during heat waves. This is important, as acute tolerance to extreme temperatures and chronic tolerance to very high temperatures may be traded off against each other (Castañeda et al., 2015; Johansson & Laurila, 2017; Rezende et al., 2014; Truebano et al., 2018; but see Jørgensen, Malte, & Overgaard, 2019). A notable exception comes from recent work on the lizard *Anolis cristatellus*, whereby daily brief exposure of embryos to extreme high temperatures reduced survival, but to the same degree in forest and urban populations (Hall & Warner, 2018). Because of the more intense and frequent heat waves in cities (Ward et al., 2016; Wouters et al., 2017; Zhao et al., 2018), we expected urban populations to have evolved a higher ability to cope with the typical chronic exposure to heat wave temperatures. In support of this, the urban larvae did not suffer a lowered energy storage and showed a smaller reduction (nonsignificant trend) in net energy budget, CEA, in response to the heat wave as compared to the rural larvae. Stressful conditions such as exposure to extreme temperatures may also cause anaerobic metabolism to be activated in order to compensate for the increased demand for energy (Pörtner, Bock, & Mark, 2017; Sokolova, 2013; Verberk, Sommer, Davidson, & Viant, 2013), a pattern also documented in damselflies (Op de Beeck, Verheyen, & Stoks, 2017). Given that metabolic capacities can evolve in response to local thermal conditions (Pörtner et al., 2017), it is possible that urban populations have evolved a more efficient anaerobic metabolism, yet we are not aware of any study testing this. Reduced energy budgets due to stress can have serious long‐term fitness consequences, including decreased growth, survival and reproduction (Sokolova, 2013). For example, decreased energy budgets in damselflies have been associated with lower growth and survival when exposed to a pesticide (Van Dievel et al., 2019; Verheyen & Stoks, 2020). Moreover, reduced energy storage may be carried over to the adult stage and is shown to result in lower mating success in *Coenagrion* damselflies (Therry, Gyulavári, Schillewaert, Bonte, & Stoks, 2014).

By integrating relevant key environmental stressors related to urbanization (i.e., a heat wave and ectoparasites) in a common‐garden experiment as advocated by Rivkin et al. (2019), we were able to identify likely genetically based physiological differentiation between urban and rural populations. Notably, this urban–rural differentiation in the bioenergetic variables would have remained undetected if we had not exposed the larvae to the heat wave. Increasing rates of urbanization and global warming are expected to cause more frequent, longer and more intense heat waves (Meehl & Tebaldi, 2004; Stillman, 2019; Wang & Dillon, 2014; Ward et al., 2016; Wouters et al., 2017). Importantly, changes in these parameters, that is the frequency, duration and amplitude of exposure to extreme temperatures, can have severe fitness consequences (e.g., Fischer et al., 2014; Hall & Warner, 2018; Ma, Rudolf, & Ma, 2015; Sentis, Hemptinne, & Brodeur, 2013). Hence, exposure to more severe (e.g., longer than the here applied four‐day period, with peak temperatures > 32°C) and more frequent heat wave periods would likely exacerbate the detrimental bioenergetic effects reported for immune‐challenged rural larvae. This would leave them with even more limited resources that can be allocated to energetically costly stress coping mechanisms such as antioxidant defence and immune function (González‐Tokman et al., 2020; Lee, 2006; Monaghan, Metcalfe, & Torres, 2009). Given that heat waves experienced during early stages can carry‐over and reduce fitness in later stages (e.g., Zhang et al., 2015) and even alter biotic interactions (Stoks, Verheyen, Van Dievel, & Tüzün, 2017), the here reported higher ability to cope with heat waves may be fundamental for the survival of urban damselfly populations in the stressful city environment.

## CONFLICT OF INTEREST

We declare no conflict of interest.

## Supporting information

Appendix S1

Appendix S2

Appendix S3

## Data Availability

Data used in this study are archived at Figshare: https://doi.org/10.6084/m9.figshare.12799958.
