# Supplementary material for: Lower bioenergetic costs but similar immune responsiveness under a heat wave in urban compared to rural damselflies
Source: Evol Appl. 2020 Jul 9;14(1):24–35. doi: 10.1111/eva.13041 (PMC7819556; doi:10.1111/eva.13041)
Supplement: Supplementary file 1 — Appendix S1 [file EVA-14-24-s001.docx]

**Appendix S1:** Prevalence of parasitism by water mites of rural and urban adult damselflies

**Lower bioenergetic costs but similar immune responsiveness under a heat wave in urban compared to rural damselflies**

Between 1 – 29 July 2013, as part of another study, we collected adult male *Coenagrion puella* damselflies (rural N =207, urban N = 229) from the same six populations (as described in the main text), and counted the number of water mites attached to each individual using a magnifying glass. Water mites are important ectoparasites of adult damselflies, including *C. puella* (Rolff, 2000), and may negatively affect damselfly host survival and mating success (Forbes, 1991, 1993; Forbes & Baker, 1991). Water mites attach to late instar larvae, and feed on the body fluids of the damselfly both during larval and adult stages (Yourth et al., 2001). Damselflies defend against mite parasitism by encapsulating the stylostome (feeding tube) of the water mite with layers of melanin, which has been shown to result in the death of the water mite (Yourth et al., 2001).

To statistically test for urbanisation-related differences in the number of water mites found on adult damselflies, we used a zero-inflated model with a negative binomial error term (Zuur et al., 2009). This approach was chosen due to overdispersion and the high frequency of zeros in our data (see Table X and Fig. X in Appendix). We used the R package ‘*pscl’* for running the zero-inflated negative binomial model (Zeileis et al., 2008).

**Results**

The mean number of water mites found on adult damselflies from rural populations (mean ± SE = 0.039 ± 0.017) was lower than on the ones from urban populations (0.266 ± 0.182) (likelihood ratio test, χ^2^_2_ = 8.37, p = 0.0152, see also Fig. S1).

**Figure S1.** Number of water mites found on adult *Coenagrion puella* damselflies across rural and urban populations. The figures, and accompanying summary tables, illustrate the number of water mites for (a) the full dataset and (b) the dataset excluding data points with zero water mites.

**References**

Forbes, M. R. L. (1991). Ectoparasites and mating success of male *Enallagma ebrium* damselflies (Odonata: Coenagrionidae). *Oikos*, *60*(3), 336. <https://doi.org/10.2307/3545076>

Forbes, M. R. L. (1993). Parasitism and host reproductive effort. *Oikos*, *67*(3), 444. <https://doi.org/10.2307/3545356>

Forbes, M. R. L., & Baker, R. L. (1991). Condition and fecundity of the damselfly, *Enallagma ebrium* (Hagen): the importance of ectoparasites. *Oecologia*, *86*(3), 335–341. <https://doi.org/10.1007/BF00317598>

Rolff, J. (2000). Water mite parasitism in damselflies during emergence: two hosts, one pattern. *Ecography*, *23*(3), 273–282. <https://doi.org/10.1034/j.1600-0587.2000.d01-1635.x>

Yourth, C. P., Forbes, M. R., & Smith, B. P. (2001). On understanding variation in immune expression of the damselflies *Lestes* spp. *Canadian Journal of Zoology*, *79*(5), 815–821. <https://doi.org/10.1139/z01-044>

Zeileis, A., Kleiber, C., & Jackman, S. (2008). Regression models for count data in R*. Journal of Statistical Software*, 27(8), 1–25. https://doi.org/10.18637/jss.v027.i08

Zuur, A. F., Ieno, E. N., Walker, N., Saveliev, A. A., & Smith, G. M. (2009). Mixed effects models and extensions in ecology with R. Springer New York, New York, USA.
