# Supplementary material for: Lower bioenergetic costs but similar immune responsiveness under a heat wave in urban compared to rural damselflies
Source: Evol Appl. 2020 Jul 9;14(1):24–35. doi: 10.1111/eva.13041 (PMC7819556; doi:10.1111/eva.13041)
Supplement: Supplementary file 2 — Appendix S2 [file EVA-14-24-s002.docx]

**Appendix S2:** Details of physiological analyses

**Lower bioenergetic costs but similar immune responsiveness under a heat wave in urban compared to rural damselflies**

For the quantification of the energy reserves, we measured the total fat, protein and sugar contents. We homogenised each larvae using a pestle and diluted the homogenate 15 times in phosphate buffer saline. The sample was centrifuged for 7 min (8000 g, 4 °C). We took 35 µl of the resulting supernatant and diluted this three times in Milli-Q water. We quantified the fat content based on the protocol of Bligh & Dyer (1959). We filled a 2-ml glass tube with a mixture of 8 µl of the supernatant and 56 µl sulphuric acid (100 %). The tubes were heated for 20 min at 150 °C. After cooling down the sample at room temperature, we added 64 µl Milli-Q water, and the whole sample was mixed. We filled a transparent 384-well microtiter plate with 30 µl of the sample and measured absorbance at 490 nm (in triplicate). Fat contents were calculated using a standard curve of glyceryl tripalmitate. We measured the protein content based on the Bradford (1976) method. We added 160 µl Milli-Q water and 1 µl of the supernatant to a 96-well microtiter plate. Then we added 40 µl Biorad protein dye and mixed the sample. We incubated the plate for 5 min at 25 °C and subsequently measured absorbance at 595 nm (in quadruplicate). The protein content was calculated based on a standard curve of known protein concentrations. The sugar content (glucose and glycogen: Hahn & Denlinger, 2007) was measured using a protocol based on the glucose kit of Sigma-Aldrich USA (Stoks et al., 2006). A mixture of 5 µL homogenate, 13 µL PBS, and 2 µL amyloglucosidase was added to the wells of a 96 well microtiter plate. After an incubation period of 30 minutes at 37 °C, all glycogen was transformed to glucose and then 40 µL glucose assay reagent was added to each well. After a final 20-minute incubation at 30 °C, absorbance was measured at 340 nm. A standard curve based on known glucose concentrations and absorbances was used to calculate sugar contents.

We measured the activity of the electron transport system (ETS) to assess metabolic rate by using the protocol of De Coen & Janssen (2003), which was modified for damselflies (Janssens & Stoks, 2013). A mixture of 5 µL supernatant and 15 µL buffered substrate solution (0.13 M Tris-HCl, 0.3% Triton X-100, 1.7 mM NADH, 250 µM NADPH, pH 8.5) was loaded in duplicate to wells of a 384-well microtiter plate. Then, 10 µL iodonitrotetrazolium (INT, 8 mM p-iodonitrotetrazolium) was added to replace O_2_ as electron acceptor and to receive electrons from NADPH via NADH-cytochrome oxidoreductase, causing the formation of formazan. The increase in formazan absorbance was measured at 20 °C, every 30 seconds during 5 minutes at 490 nm. By using the law of Lambert-Beer and a molecular extinction coefficient of 15.9 mM^-1^cm^-1^, formazan concentrations were calculated. Finally, we calculated cellular oxygen consumption rates based on the stoichiometric relationship that 1 µmol O_2_ is used to form 2 µmol formazan in the ETS complex. ETS activity was expressed as nmol O_2_ per minute per larva.

**References**

Bligh, E. G., & Dyer, W. J. (1959). A rapid method of total lipid extraction and purification. *Canadian Journal of Biochemistry and Physiology*, *37*(8), 911–917. <https://doi.org/10.1139/o59-099>

Bradford, M. M. (1976). A rapid and sensitive method for the quantitation of microgram quantities of protein utilizing the principle of protein-dye binding. *Analytical Biochemistry*, *72*(1–2), 248–254. <https://doi.org/10.1016/0003-2697(76)90527-3>

De Coen, W. M., & Janssen, C. R. (2003). The missing biomarker link: Relationships between effects on the cellular energy allocation biomarker of toxicant-stressed *Daphnia magna* and corresponding population characteristics. *Environmental Toxicology and Chemistry*, *22*(7), 1632–1641. <https://doi.org/10.1002/etc.5620220727>

Hahn, D. A., & Denlinger, D. L. (2007). Meeting the energetic demands of insect diapause: Nutrient storage and utilization. *Journal of Insect Physiology*, 53(8), 760–773. https://doi.org/10.1016/j.jinsphys.2007.03.018

Janssens, L., & Stoks, R. (2013). Synergistic effects between pesticide stress and predator cues: Conflicting results from life history and physiology in the damselfly *Enallagma cyathigerum*. *Aquatic Toxicology*, *132*–*133*, 92–99. <https://doi.org/10.1016/j.aquatox.2013.02.003>

Stoks, R., De Block, M., & McPeek, M. A. (2006). Physiological costs of compensatory growth in a damselfly. *Ecology*, *87*(6), 1566–1574. https://doi.org/10.1890/0012-9658(2006)87[1566:PCOCGI]2.0.CO;2
