# Supplementary material for: Lower bioenergetic costs but similar immune responsiveness under a heat wave in urban compared to rural damselflies
Source: Evol Appl. 2020 Jul 9;14(1):24–35. doi: 10.1111/eva.13041 (PMC7819556; doi:10.1111/eva.13041)
Supplement: Supplementary file 3 — Appendix S3 [file EVA-14-24-s003.docx]

**Appendix S3:** Additional tables

**Lower bioenergetic costs but similar immune responsiveness under a heat wave in urban compared to rural damselflies**

**Table S1.** Pairwise Pearson’s correlations, and associated p-values, between growth rate, encapsulation response, and energy budget traits (d.f. = 220 for all pairwise tests). Correlations that remained significant (p < 0.05) after the Bonferroni-correction are indicated in bold.

|  | Growth rate | Encapsulation response | Energy availability | Energy consumption |
| --- | --- | --- | --- | --- |
| Encapsulation response | **r = -0.202**  **p = 0.002** |  |  |  |
| Energy availability | r = -0.159  p = 0.018 | **r = -0.195**  **p = 0.003** |  |  |
| Energy consumption | r = -0.061  p = 0.368 | r = 0.156  p = 0.019 | r = -0.167  p = 0.012 |  |
| Cellular energy allocation | r = 0.140  p = 0.037 | **r = -0.232**  **p < 0.001** | **r = 0.726**  **p < 0.001** | **r = -0.781**  **p < 0.001** |

**Table S2.** Results of linear mixed-effect models testing for effects of heat wave, urbanization, and their interaction on growth rate, encapsulation response, energy availability, energy consumption, and cellular energy allocation in the larvae of *Coenagrion puella* damselflies. Body mass and larval age (log-transformed) were included as covariates in certain models.

|  | Growth rate | | | Encapsulation response | | |
| --- | --- | --- | --- | --- | --- | --- |
|  | F | df | P | F | df | P |
| Heat wave (HW) | 28.73 | 1,215.9 | < 0.0001 | 47.95 | 1,215.1 | < 0.0001 |
| Urbanization (URB) | 0.10 | 1,4.1 | 0.7731 | 0.28 | 1,4.1 | 0.6221 |
| HW × URB | 1.02 | 1,215.6 | 0.3140 | 0.08 | 1,214.6 | 0.7722 |
| body mass | - | - | - | 0.05 | 1,215.5 | 0.8790 |
| age (log) | 3.60 | 1,217 | 0.0592 | 0.02 | 1,215.9 | 0.8160 |

**Table S2.** (continued)

|  | Energy availability | | | Energy consumption | | | Cellular energy allocation | | |
| --- | --- | --- | --- | --- | --- | --- | --- | --- | --- |
|  | F | df | P | F | df | P | F | df | P |
| Heat wave (HW) | 15.22 | 1,213 | 0.0001 | 38.09 | 1,215.1 | < 0.0001 | 45.17 | 1,213.9 | < 0.0001 |
| Urbanization (URB) | 0.57 | 1,4 | 0.4930 | 0.45 | 1,4.1 | 0.5397 | 1.15 | 1,4 | 0.3441 |
| HW × URB | 5.05 | 1,212.8 | 0.0257 | 0.54 | 1,214.6 | 0.4648 | 2.97 | 1,213.6 | 0.0863 |
| body mass | 0.11 | 1,213.6 | 0.7413 | 30.18 | 1,215.5 | < 0.0001 | 12.56 | 1,214.6 | 0.0005 |
| age (log) | 2.52 | 1,214.3 | 0.1141 | 0.16 | 1,215.9 | 0.6901 | 1.90 | 1,215.3 | 0.1693 |

**Table S3.** Results of linear mixed-effect models testing for effects of heat wave, urbanization, and their interaction on fat, protein, and sugar content, and activity of the electron transport system (ETS) in the larvae of *Coenagrion puella* damselflies. Body mass and larval age (log-transformed) were included as covariates.

|  | Fat | | | Protein | | |
| --- | --- | --- | --- | --- | --- | --- |
|  | F | df | P | F | df | P |
| Heat wave (HW) | 14.027 | 1,213 | 0.0002 | 0.0489 | 1,215.1 | 0.8250 |
| Urbanization (URB) | 0.5763 | 1,4 | 0.4900 | 0.0118 | 1,4.1 | 0.9185 |
| HW × URB | 3.1029 | 1,212.9 | 0.0796 | 0.198 | 1,214.6 | 0.6568 |
| body mass | 16.283 | 1,213.7 | 0.0001 | 212.582 | 1,215.5 | < 0.0001 |
| age (log) | 4.5279 | 1,214.3 | 0.0345 | 5.1131 | 1,215.9 | 0.0247 |

**Table S3.** (continued)

|  | Sugar | | | ETS | | |
| --- | --- | --- | --- | --- | --- | --- |
|  | F | df | P | F | df | P |
| Heat wave (HW) | 0.9423 | 1,213.9 | 0.3330 | 37.3904 | 1,215.1 | < 0.0001 |
| Urbanization (URB) | 1.2368 | 1,4 | 0.3281 | 0.3634 | 1,4.1 | 0.5787 |
| HW × URB | 1.0034 | 1,213.6 | 0.3176 | 0.3563 | 1,214.6 | 0.5512 |
| body mass | 12.491 | 1,214.6 | 0.0005 | 135.6693 | 1,215.5 | < 0.0001 |
| age (log) | 18.0062 | 1,215.3 | < 0.0001 | 0.0857 | 1,215.9 | 0.7700 |
